# A Patch and Attention Mechanism-Based Model for Multi-Parameter Prediction of Rabbit House Environmental Parameters

**DOI:** 10.3390/ani15213192

**Published:** 2025-11-02

**Authors:** Ronghua Ji, Guoxin Wu, Hongrui Chang, Zhongying Liu, Zhonghong Wu

**Affiliations:** 1 College of Information and Electrical Engineering, China Agricultural University, Beijing 100083, China; jessic1212@cau.edu.cn (R.J.); wugx22@163.com (G.W.); s20233081774@cau.edu.cn (H.C.); 2 State Key Laboratory of Animal Nutrition and Feeding, College of Animal Science and Technology, China Agricultural University, Beijing 100093, China; lzy228@cau.edu.cn

**Keywords:** rabbit, environmental parameters, time-series prediction, patching mechanism, attention mechanism, environmental regulation

## Abstract

**Simple Summary:**

Accurate prediction of temperature, humidity, and carbon dioxide concentration inside rabbit houses makes it possible to regulate the environment more precisely, which improves housing conditions and ultimately enhances the health and productivity of rabbits. To improve prediction accuracy and cross-regional generalization, a multi-parameter prediction model for rabbit houses is proposed, integrating patching and attention mechanisms. The model employs differentiated encoding strategies for environmental and auxiliary parameters, substantially enhancing its multi-parameter modeling capabilities. Experimental results indicate that the model achieves superior predictive accuracy and strong cross-regional generalization, supporting intelligent environmental regulation in intensive rabbit farming.

**Abstract:**

The health and productivity of rabbits are highly sensitive to the environmental conditions within the rabbit house, particularly to fluctuations and deviations in temperature, relative humidity, and carbon dioxide (CO_2_) concentration. However, owing to the thermal inertia and residual evaporation effects inherent in ventilation and cooling systems, environmental changes often exhibit delayed responses, rendering real-time control inadequate. Accurate prediction of key environmental parameters is indispensable for formulating effective environmental control strategies, as it enables consideration of their future dynamics and thereby enhances the rationality of regulation in rabbit farming. Existing prediction models often exhibit unsatisfactory accuracy and weak generalization, which restricts the incorporation of prediction into effective environmental control strategies. To address these limitations, summer indoor and outdoor environmental data were collected from rabbit houses in Nanping, Fujian; Jiyuan, Henan; and Qingyang, Gansu, China—three climatically distinct regions—forming three datasets. Based on these datasets, a multi-parameter time-series prediction model, Patch and Cross-Attention Enhanced Transformer for Rabbit House Prediction (PatchCrossFormer-RHP), is introduced, integrating patching and attention mechanisms. The model partitions the sequences of rabbit house temperature, relative humidity, and CO_2_ concentration into patches and incorporates auxiliary parameters, such as indoor air velocity and outdoor temperature and humidity, to enhance feature representation. Furthermore, it applies cross-attention with differentiated encoding to disentangle multi-parameter relationships and improve predictive performance. This study used the Fujian dataset as the primary benchmark. On this dataset, PatchCrossFormer-RHP achieved root mean square error (RMSE) values of 0.290 °C, 1.554%, and 38.837 ppm for rabbit house temperature, humidity, and CO_2_ concentration, respectively, with corresponding R^2^ values of 0.963, 0.956, and 0.838, consistently outperforming RNN, GRU, and LSTM. Transfer experiments with single- and multi-source pretraining followed by fine-tuning on Fujian demonstrated that strong cross-regional generalization can be achieved with only limited target-domain data.

## 1. Introduction

In recent years, large-scale rabbit farming in China has experienced rapid development. From 2021 to 2023, the rabbit industry index continued to rise, and rabbit meat production reached 458,000 tons in 2023 [1]. Intensive farming systems contribute to the sustainable development of the rabbit industry. However, under enclosed and high-density conditions, improper regulation of environmental factors—such as temperature, humidity, and harmful gases—can easily induce stress responses and diseases, thereby impairing rabbit growth and development [2,3,4]. The core of intelligent environmental regulation systems in rabbit houses lies in the control algorithm, which dynamically adjusts the operating states of fans, cooling pads, and heaters according to real-time environmental parameters to maintain optimal housing conditions. Existing algorithms typically rely only on current parameter values and lack the ability to anticipate future trends, often resulting in insufficient regulation accuracy [5,6,7]. However, environmental regulation in rabbit houses often exhibits time-lag effects caused by thermal inertia and residual evaporation, which delay the system’s response to control actions. These lag characteristics make real-time control insufficient, highlighting the necessity of predictive modeling to anticipate and compensate for environmental fluctuations. In addition, integrating prediction results with control algorithms enables proactive adjustment of ventilation, cooling, heating, and other components of the environmental regulation system, thereby improving overall stability and efficiency. Therefore, accurate prediction of indoor environmental parameters is essential for designing and implementing effective regulation strategies, improving control precision, and ultimately enhancing rabbit comfort and animal welfare [8,9].

Prediction models for livestock and poultry housing environments can be broadly categorized into physics-based statistical models, machine learning models, and deep learning models. Physics-based statistical models rely heavily on precise experimental data and extensive domain expertise, making them challenging to construct and limiting their transferability [10,11,12]. Machine learning models, such as those based on support vector regression (SVR) [13] and extreme learning machines (ELM) [14], adopt data-driven approaches to capture the coupling relationships among environmental variables, thereby enhancing model applicability and flexibility. However, these models remain highly dependent on data quality, while livestock housing data are often susceptible to noise interference. Both physics-based and machine learning models show limited capability in feature extraction and nonlinear modeling. By contrast, deep learning models, with their powerful capabilities for automatic feature learning and nonlinear modeling, have emerged as a key research direction for predicting environmental parameters in livestock housing [15,16,17,18,19,20,21,22]. For instance, Lee [22] employed recurrent neural networks (RNNs) to predict temperature and humidity in duck houses under different ventilation modes. Li [21] compared RNN, LSTM, and GRU for predicting dissolved oxygen in fish ponds, demonstrating that LSTM and GRU achieved superior performance, with GRU requiring fewer parameters. Liu [23] further demonstrated that Transformer-based models achieved higher accuracy and stability than mainstream architectures, such as BERT and Seq2Seq, in rabbit house environmental prediction tasks.

However, the strong interdependence among environmental factors in livestock and poultry houses makes it challenging to ensure prediction accuracy. Feng [24] applied principal component analysis (PCA) to reduce the number of environmental parameters in sheep houses, which effectively improved prediction accuracy. Yang [25] proposed an empirical mode decomposition (EMD)-based model for predicting ammonia concentration in pig houses, enhancing both accuracy and efficiency. Ji [26] employed the seasonal-trend decomposition procedure (STL) to decompose rabbit house environmental sequences into trend, seasonal, and residual components, thereby partially addressing the problem of variable coupling. These studies primarily focused on data preprocessing and feature extraction, showing that sequence decomposition can improve predictive accuracy. Nevertheless, when applied to different regions or different types of livestock housing, the generalization ability of existing models remains limited. In particular, there is a lack of discussion on cross-regional deployment and insufficient analysis of model adaptability for heterogeneous housing environments.

In summary, rabbit house environment prediction faces challenges, such as limited accuracy and generalization in multi-parameter and multi-step forecasting, and a lack of research on cross-regional model deployment. To address these challenges, this study aims to develop a unified prediction framework, termed PatchCrossFormer-RHP (Patch and Cross-Attention Enhanced Transformer for Rabbit House Prediction), by enhancing Transformer-based architectures through the integration of patch-based temporal encoding and a cross-attention mechanism. This framework is designed to achieve high-precision and generalizable forecasting of key indoor environmental parameters—temperature, relative humidity, and CO_2_ concentration—in rabbit houses across different regions.

## 2. Materials and Methods

### 2.1. Data Collection

Environmental parameters were collected from three enclosed rabbit houses located in Qingyang (Gansu, China), Jiyuan (Henan, China), and Nanping (Fujian, China). These farms are situated in distinct climatic zones, representing the northwest arid, central temperate, and southeastern humid regions of China’s rabbit-farming areas. Each site was an intensive rabbit house with clear environmental regulation needs, selected to ensure data representativeness and reliability. This inter-regional design emphasizes climatic diversity to validate the model’s cross-regional transferability.

**Figure 1:**
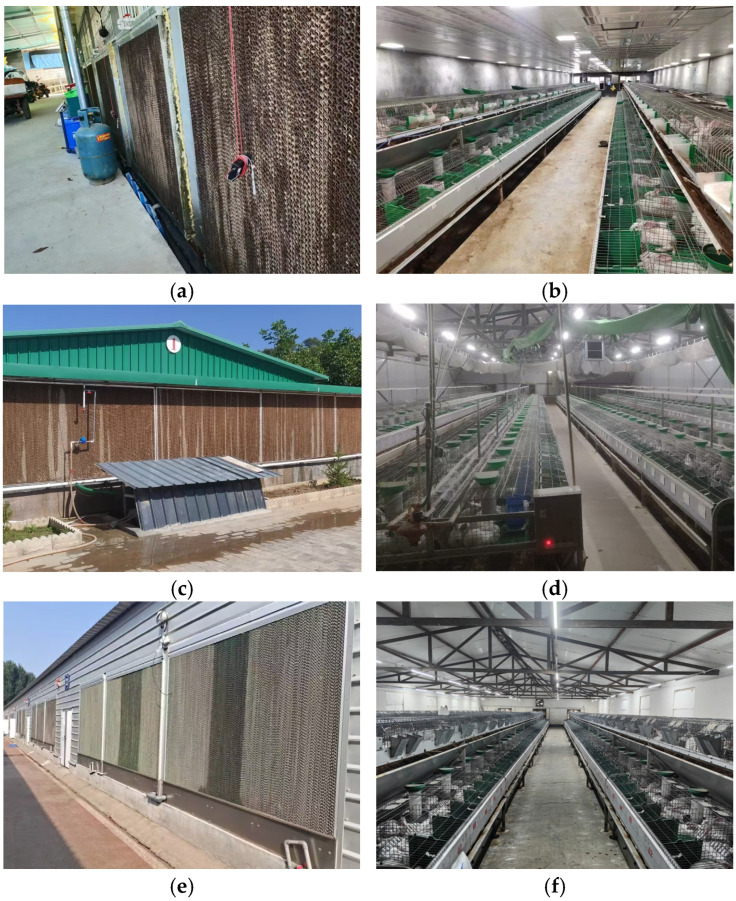
Actual exterior and interior views of the rabbit houses. (**a**) Exterior view of the rabbit house in Nanping, Fujian; (**b**) Interior view of the rabbit house in Nanping, Fujian; (**c**) Exterior view of the rabbit house in Qingyang, Gansu; (**d**) Interior view of the rabbit house in Qingyang, Gansu; (**e**) Exterior view of the rabbit house in Jiyuan, Henan; (**f**) Interior view of the rabbit house in Jiyuan, Henan.

Table 1 summarizes the characteristics of the rabbit houses, and Figure 1 illustrates the housing conditions.

In addition to indoor sensors, outdoor sensors were mounted on shaded exterior walls to record air temperature and humidity. These parameters serve as external drivers influencing indoor thermal and moisture dynamics. The same type and number of sensors were installed in all three rabbit houses. The sensor specifications are listed in Table 2.

**Figure 2:**
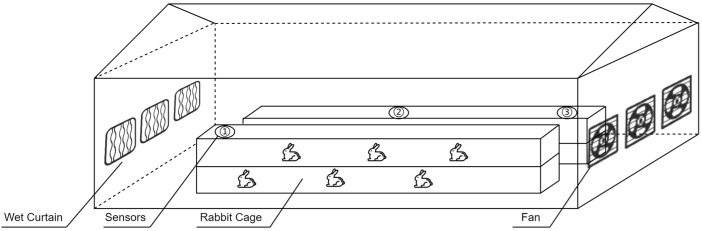
Schematic Diagram of Indoor Sensor Deployment. The sensors are divided into three groups, each consisting of a temperature and humidity sensor, a CO_2_ concentration sensor, and a wind speed sensor. Three sets of sensors were arranged laterally within each rabbit house: Group ① was positioned near the wet curtain side, Group ② at the center, and Group ③ near the fan side. The outdoor sensor was installed on a shaded section of the exterior wall.

To ensure that the collected data accurately reflected the actual environment of the rabbit houses, the sensors inside the houses were arranged as shown in Figure 2.

The data collection periods were as follows: Nanping, Fujian (1 August–30 September 2023); Qingyang, Gansu (25 July–30 September 2023); and Jiyuan, Henan (12 August–30 September 2023). Summer was chosen as the sampling period because it represents the most heat-stress-prone season, when environmental regulation faces the highest demand for intelligent control. The sampling frequency was set to once per minute.

### 2.2. Data Preprocessing

During data collection, occasional sensor errors resulted in localized anomalies, manifested as abnormally high or low values at specific time points. To address this, outlier detection was first performed using a sliding-window method to identify abnormal points, which were then marked and set as null values. Additionally, data loss occurred due to external factors, including network failures or temporary power outages. For these outliers and missing values (with missing durations not exceeding 60 min), linear interpolation was uniformly applied for imputation. The calculation method of linear interpolation is given in Equation (1):

(1) $$y_{k}=y_{1}+\frac{k}{n+1}\cdot \left(y_{2}-y_{1}\right),k=1,2,\ldots ,n$$

$y_{1}$,$y_{2}$ represent the nearest valid values before and after the missing interval, respectively. n denotes the total number of missing values, and $y_{k}$ represents the interpolated value for the k-th missing data point.

The sampling frequency was set to once per minute, while the environmental parameters in rabbit houses generally exhibited slow variation. To improve model performance, a sliding-average method was applied for down-sampling, adjusting the data interval to 10 min. This process effectively smoothed local fluctuations in the sequences and aligned better with practical requirements for control and decision-making.

To eliminate the effects of dimensional differences among environmental parameters and to accelerate model convergence, the data were normalized using the min–max method, as defined in Equation (2):

(2) $$x_{scaled}^{i}=\frac{x^{i}-x_{min}^{i}}{\left(x_{max}^{i}-x_{min}^{i}\right)+10^{-7}}$$

$x_{scaled}^{i}$ denotes the normalized value of the environmental parameter $x^{i}$, while $x_{min}^{i}$ and $x_{max}^{i}$ represent the minimum and maximum values of the i-th parameter, respectively.

### 2.3. Data Analysis

The environmental parameters in rabbit houses exhibit nonlinear, non-stationary, periodic, and strongly coupled characteristics [27]. Based on a detailed comparative analysis of the environmental features and differences across the three regions during the sampling period, data from one representative week (20–26 August 2023) were selected for illustrative presentation. This period corresponds to the late summer season, one of the most complex transitional phases of the year, during which climatic differences among the three regions are most pronounced. For each region, the outdoor temperature and humidity data collected during this week were aligned by time points. The mean values of the observations at the same time points across seven days were calculated, and their maximum, minimum, and diurnal ranges were further derived. The results are presented in Table 3.

It can be observed that notable differences exist in the outdoor temperature and humidity among the three regions. In Nanping, Fujian, both daytime temperature and humidity were higher than those in Qingyang, Gansu, and Jiyuan, Henan, with smaller diurnal variations. Qingyang, Gansu, recorded the lowest temperature and humidity, accompanied by pronounced diurnal fluctuations. The conditions in Jiyuan, Henan, were intermediate between those of Fujian and Gansu, showing relatively strong variability.

**Figure 3:**
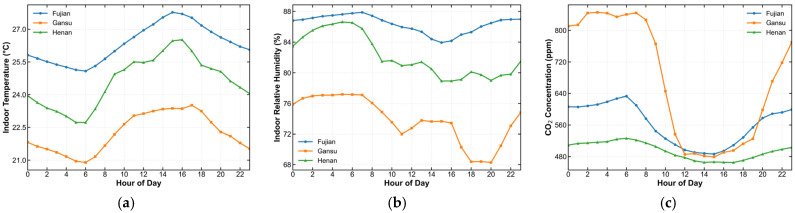
Seven-day mean variation curves of indoor temperature, humidity, and CO_2_ concentration in the three regions. (**a**) Temperature; (**b**) Humidity; (**c**) CO_2_ concentration.

Similarly, the seven-day mean variation curves of indoor temperature, humidity, and CO_2_ concentration for the three regions were computed, and the results are illustrated in Figure 3.

As shown in Figure 3, the temperature and humidity in the Fujian rabbit house were significantly above the optimal ranges (the recommended environmental temperature for rabbits is typically 15–25 °C, and the optimal relative humidity is 60–65% [28]), indicating a potential risk of heat stress. In contrast, the Gansu rabbit house maintained temperatures close to the suitable range but exhibited large diurnal fluctuations, while CO_2_ concentrations remained persistently high, suggesting insufficient nighttime ventilation. The Henan rabbit house exhibited intermediate temperature and humidity levels compared to the other two sites, with relatively high variability. Overall, the three rabbit houses displayed marked differences in their environmental parameters.

**Figure 4:**
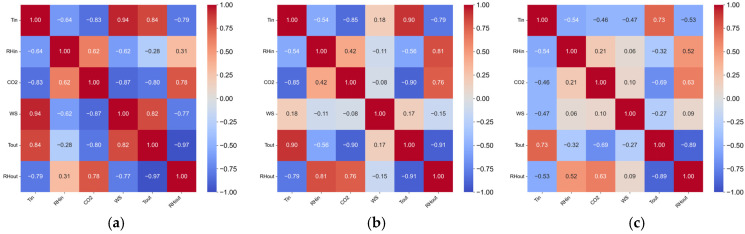
Correlation heatmaps of environmental parameters in rabbit houses. (**a**) Fujian; (**b**) Gansu; (**c**) Henan. Tin: indoor temperature; RHin: indoor humidity; CO_2_: indoor CO_2_ concentration; WS: indoor wind speed; Tout: outdoor temperature; RHout: outdoor humidity.

To evaluate the coupling strength and structural differences among different parameters, Pearson correlation coefficients were calculated for all environmental variables. The correlation heatmaps of rabbit house parameters in the three regions are presented in Figure 4.

It can be observed that indoor temperature, indoor humidity, and indoor CO_2_ concentration were strongly coupled with each other, while indoor wind speed, outdoor temperature, and outdoor humidity exerted significant influences on these three indoor parameters. Moreover, the correlations among environmental variables varied across the three regions. The coupling was strongest in Fujian, whereas the parameters in Gansu appeared relatively independent of each other. These differences can be attributed to regional variations in climate conditions, housing structures, stocking densities, management practices, and environmental control systems.

### 2.4. Predictive Model Construction

Based on the analysis of environmental parameters in rabbit houses and considering the coupling relationships among variables, we propose PatchCrossFormer-RHP (Patch and Cross-Attention Enhanced Transformer for Rabbit House Prediction) to improve prediction accuracy, stability, and model generalization.

PatchCrossFormer-RHP adopts a modeling strategy that integrates both temporal and channel dimensions. The model input is divided into two parts: the environmental parameter sequence and the auxiliary parameter sequence. The environmental parameter sequence includes indoor temperature, indoor humidity, and indoor CO_2_ concentration, while the auxiliary parameter sequence consists of indoor wind speed, outdoor temperature, and outdoor humidity. The model outputs are the predicted sequences of indoor temperature, humidity, and CO_2_ concentration.

**Figure 5:**
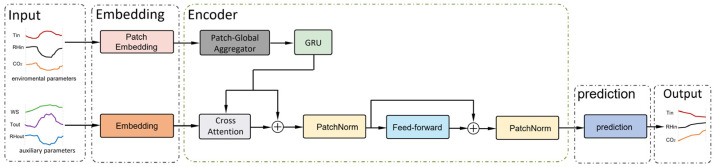
Architecture of PatchCrossFormer-RHP. The model includes an embedding layer, an encoder layer, and a prediction layer to output the predicted sequences of indoor temperature, humidity, and CO_2_ concentration. Tin, RHin, CO_2_, WS, Tout, and RHout denote indoor temperature, indoor humidity, indoor CO_2_ concentration, indoor wind speed, outdoor temperature, and outdoor humidity, respectively.

PatchCrossFormer-RHP consists of an embedding layer, an encoder layer, and a prediction layer. The overall architecture is illustrated in Figure 5.

#### 2.4.1. Embedding Layer

The embedding layer encodes the input environmental and auxiliary parameter sequences, projecting them into continuous vector representations that serve as inputs to subsequent modules.

In time-series forecasting models, two primary encoding strategies are commonly used: time dimension encoding and channel dimension encoding. These approaches enhance the model’s ability to capture temporal dynamics from different perspectives. Time dimension encoding improves the model’s sensitivity to local trends (e.g., short-term fluctuations) and global patterns (e.g., long-term cycles) within sequences. In contrast, channel dimension encoding captures the coupling relationships among different input variables. PatchCrossFormer-RHP adopts a modeling strategy that integrates both encoding types.

The model receives three environmental parameter sequences *X* and four auxiliary parameter sequences *S*, as defined in Equations (3) and (4):

(3) $$X=\{x_{1},x_{2},x_{3}\}$$

(4) $$S=\{s_{1},s_{2},s_{3}\}$$

$x_{i}{,s}_{i}\in R^{N}$, $x_{1}$, $x_{2}$ and $x_{3}$ represent indoor temperature, indoor humidity, and indoor CO_2_ concentration, respectively, while $s_{1}$, $s_{2}$ and $s_{3}$ correspond to indoor wind speed, outdoor temperature, and outdoor humidity. *N* denotes the sequence length.

The environmental parameter sequence *X* is encoded using a patching strategy. Each sequence is divided into *k* non-overlapping patches of equal length. The value of *k* is determined according to Equation (5):

(5) $$k=\frac{N}{L},$$

**Figure 6:**
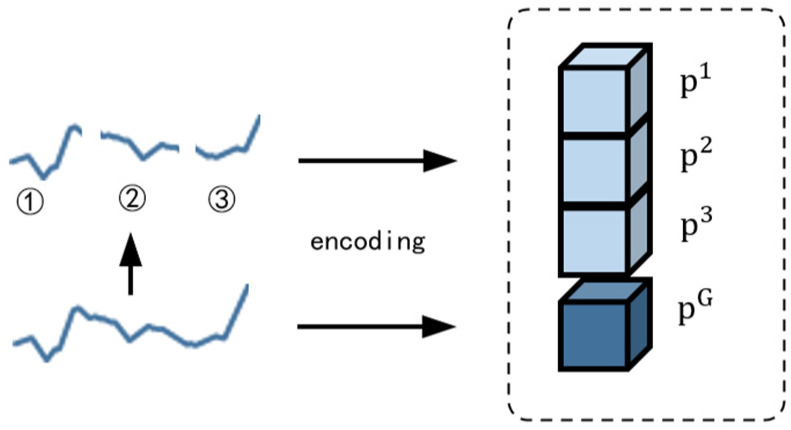
Temporal encoding strategy for environmental parameter sequences. The original sequence is divided into three segments (①–③), each representing a temporal patch. These patches are individually encoded as $p^{1}$, $p^{2}$ and $p^{3}$, while the original sequence is encoded as $p^{G}$.

*N* denotes the sequence length and *L* represents the patch length. Both the *k* patches of $x_{i}$ and the original sequence $x_{i}$ are encoded separately to capture local and global temporal features, respectively. The encoding process is illustrated in Figure 6.

This patch-based encoding strategy facilitates the extraction of sufficient intra-sequence information while preserving global sequence characteristics. It helps the model capture both local trends (e.g., short-term fluctuations) and global patterns (e.g., periodic variations) within the sequence.

The embedded and encoded environmental parameter sequence is denoted as $X_{en}$, as shown in Equation (6):

(6) $$X_{en}=\{X_{en}^{1},X_{en}^{2},X_{en}^{3}\},$$

$X_{en}^{1}$, $X_{en}^{2}$ and $X_{en}^{3}$ represent the embedded and encoded sequence vectors of indoor temperature, indoor humidity, and indoor CO_2_ concentration, respectively.

The vector $X_{en}^{i}$ contains *k* + 1 encoded vectors, corresponding to the encoded subsequences and the full sequence, as shown in Equation (7):

(7) $$X_{en}^{i}=\{p^{1},p^{2},\ldots ,p^{k},p^{G}\},$$

vector $X_{en}^{i}$ contains both local and global information of the original sequence.

Each sequence $s_{i}$ in the auxiliary parameter sequence S is encoded along the channel dimension (Equation (4)).

In this study, sinusoidal positional encoding is adopted. As an effective encoding strategy for Transformer-based long-sequence prediction models, sinusoidal positional encoding enables the model to capture both local trends and global patterns in time series data without introducing additional trainable parameters. The computation of the sinusoidal positional encoding is given in Equations (8) and (9):

(8) $$p_{\left(pos,2i\right)}=sin\left(\frac{pos}{10000^{2i/d}}\right),$$

(9) $$p_{\left(pos,2i+1\right)}=cos\left(\frac{pos}{10000^{2i/d}}\right),$$

*pos* denotes the time step, *i* denotes the dimension index, and *d* represents the embedding dimension.

#### 2.4.2. Encoder

The encoder is responsible for extracting temporal features from the sequences of environmental parameters. It is composed of three key components: the Patch-Global Aggregator, Gated Recurrent Unit (GRU), and the Cross-Attention mechanism. The Patch-Global Aggregator integrates local information across individual patches, the GRU further captures temporal dependencies within the sequence, and the cross-attention mechanism enables deep interaction between target variables and auxiliary variables.

First, considering that the contributions of local and global information at each time step t may vary, the Patch-Global Aggregator applies dynamic weighting to the *k* + 1 vectors $p^{j}$ in $X_{en}^{i}$ (where *j* = 1, 2, …, *k* and *G* denotes the global vector). The detailed computation is defined in Equations (10)–(12):

(10) $$e_{t}^{j}=\tanh\left(W_{e}\left[h_{t-1};p^{j}\right]\right),$$

(11) $$\alpha_{t}^{j}=softmax\left(e_{t}^{j}\right)=\frac{exp\left(e_{t}^{j}\right)}{\sum_{i=1}^{n}\;exp\left(e_{t}^{i}\right)},$$

(12) $${\tilde{p}}_{t}^{j}=\alpha_{t}^{k}\cdot p_{t}^{j},$$

$p^{j}$ denotes a vector in $X_{en}^{i}$ (where *j* = 1, 2,…, *k* and G represents the global vector), and $p^{j}$ is the weighted vector at time *t*. $W_{e}$ is a learnable weight matrix, and $h_{t-1}$ is the hidden state at time *t* − 1 from the Gated Recurrent Unit (GRU) following the Patch-Global Aggregator.

**Figure 7:**
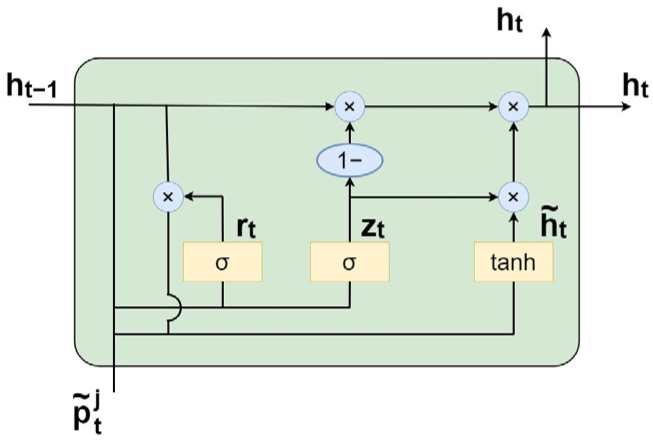
Structure of the GRU.

The GRU, an improved variant of the Recurrent Neural Network (RNN), is well-suited for temporal feature extraction due to its recurrent architecture. GRU is a computationally efficient type of RNN [29], whose core idea is to use gating mechanisms to selectively retain or update historical information, thereby capturing dynamic patterns in time series data. It consists primarily of two gates: the update gate $z_{t}$, which determines the extent to which historical and new information are integrated into the current hidden state, and the reset gate $r_{t}$, which controls how much of the past hidden state is retained when computing the candidate hidden state. The GRU structure is illustrated in Figure 7.

In the Figure 7, ${\tilde{p}}_{t}^{j}\;$represents the vector at time t after weighting by the Patch-Global Aggregator. $z_{t}$ denotes the update gate,$r_{t}$ denotes the reset gate, $h_{t-1}$ is the hidden state at time *t* − 1, $h_{t}$ is the hidden state at time *t*, and ${\tilde{h}}_{t}$ is the candidate hidden state. The definitions of the update and reset gates, as well as the hidden state update rules in GRU, are given in Equations (13)–(16):

(13) $$z_{t}=\sigma \left(W_{z}\left[h_{t-1};{\tilde{p}}_{t}^{j}\right]\right),$$

(14) $$r_{t}=\sigma \left(W_{r}\left[h_{t-1};{\tilde{p}}_{t}^{j}\right]\right),$$

(15) $${\tilde{h}}_{t}=tanh\left(W[r_{t}⊙h_{t-1};{\tilde{p}}_{t}^{j}]\right),$$

(16) $$h_{t}=(1-z_{t})⊙h_{t-1}+z_{t}⊙{\tilde{h}}_{t},$$

*σ* and *tanh* are activation functions, and $W_{z}$, $W_{r}$, and W are learnable parameter matrices.

The combination of the Patch-Global Aggregator and GRU preserves local temporal features within each patch while enhancing the model’s ability to capture global temporal dependencies across patches. This significantly enhances the model’s ability to temporally model rabbit house environmental parameters.

**Figure 8:**
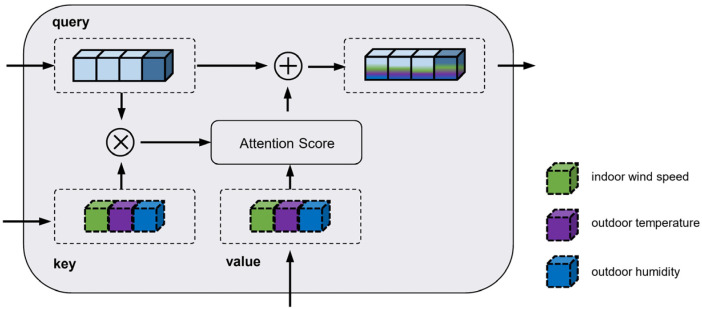
Illustration of the Cross-Attention Mechanism. Key and value are derived from encoded auxiliary parameters—indoor wind speed, outdoor temperature, and outdoor humidity.

To achieve feature decoupling between environmental and auxiliary parameters, a cross-attention mechanism is introduced. Specifically, the temporally encoded environmental parameter sequence serves as the query, while the channel-encoded auxiliary parameter sequence functions as the key and value in the attention computation. This mechanism enables the model to extract predictive and relevant information from the auxiliary sequence. The cross-attention process is illustrated in Figure 8.

The features fused through the cross-attention module are subsequently normalized to unify feature scales and accelerate training convergence. These normalized features are then passed through a feedforward neural network for nonlinear transformation.

#### 2.4.3. Prediction Layer

The prediction layer employs a target-specific modeling strategy, comprising three parallel single-layer fully connected neural networks. Each network is independently responsible for predicting one of the following target variables: temperature, humidity, or CO_2_ concentration. The number of neurons in each network equals the prediction horizon. The input to each network is formed by concatenating multiple encoded feature patches corresponding to the respective environmental parameter, which are then used to generate multi-step forecasts for that parameter.

### 2.5. Model Performance Evaluation Metrics

The model performance was evaluated using three widely adopted metrics: Mean Absolute Error (MAE), Root Mean Squared Error (RMSE), and the Coefficient of Determination (R^2^). MAE provides an absolute measure of prediction error, while RMSE emphasizes larger deviations by penalizing squared errors more heavily. R^2^ quantifies the goodness of fit between predicted and observed values, with values closer to 1 indicating better predictive accuracy. The specific calculation formulas are given in Equations (17)–(19):

(17) $$\mathrm{MAE}=\frac{1}{n}\sum_{j=1}^{n}\left|y_{j}^{\prime }-y_{j}\right|,$$

(18) $$\mathrm{RMSE}=\sqrt{\frac{1}{n}\sum_{j=1}^{n}{\left(y_{j}^{\prime }-y_{j}\right)}^{2}},$$

(19) $$R^{2}=\frac{\sum_{j=1}^{n}\;{\left(y_{j}^{\prime }-\bar{y}\right)}^{2}}{\sum_{j=1}^{n}\;{\left(y_{j}-\bar{y}\right)}^{2}},$$

*n* denotes the number of predicted values, $y_{j}^{\prime }$ and $y_{j}$ represent the predicted and actual values, respectively, and *ȳ* denotes the mean of the actual values.

### 2.6. Model Test Platform

The hardware and software environments used for model training and testing are summarized in Table 4.

The hardware and software environments employed for model training and testing are presented in Table 5.

## 3. Results

To systematically and comprehensively evaluate the performance of PatchCrossFormer-RHP in multi-parameter and multi-step prediction tasks for rabbit house environments, this study conducted analyses on the impact of input and output sequence lengths, performance evaluation experiments, and ablation studies.

The experiments were conducted using a rabbit house environmental dataset collected in Fujian Province, which contained a total of 7548 samples. The dataset was chronologically divided into training, validation, and test sets in a ratio of 6:1:3, corresponding to 4529, 755, and 2264 samples, respectively. The training set was used for model parameter learning, the validation set for hyperparameter tuning and overfitting prevention, and the test set for final performance evaluation. Each sample included both indoor environmental parameters (air temperature, relative humidity, CO_2_ concentration, and wind speed) and outdoor parameters (air temperature and relative humidity). Within a multivariate prediction framework, the model was designed to forecast three key indicators: indoor air temperature, relative humidity, and CO_2_ concentration. Wind speed and outdoor environmental variables were incorporated as auxiliary inputs, while the prediction targets were restricted to the indoor parameters of temperature, humidity, and CO_2_ concentration.

### 3.1. Analysis of the Impact of Input and Output Sequence Lengths on Prediction Performance

The lengths of input and output sequences are critical parameters of the model. The input sequence represents the historical environmental data of the rabbit houses received by the model, and its length determines the range of historical information that the model can perceive. The output sequence length corresponds to the forecasting horizon, with different horizons serving different application scenarios and response requirements.

To evaluate the effects of varying input and output sequence lengths on model performance, a total of 21 experiments were conducted. Specifically, the input sequence length was set to seven values: 6, 12, 24, 48, 72, 96, and 144. For each input length, the output sequence length was configured to three values: 6, 12, and 24.

**Figure 9:**
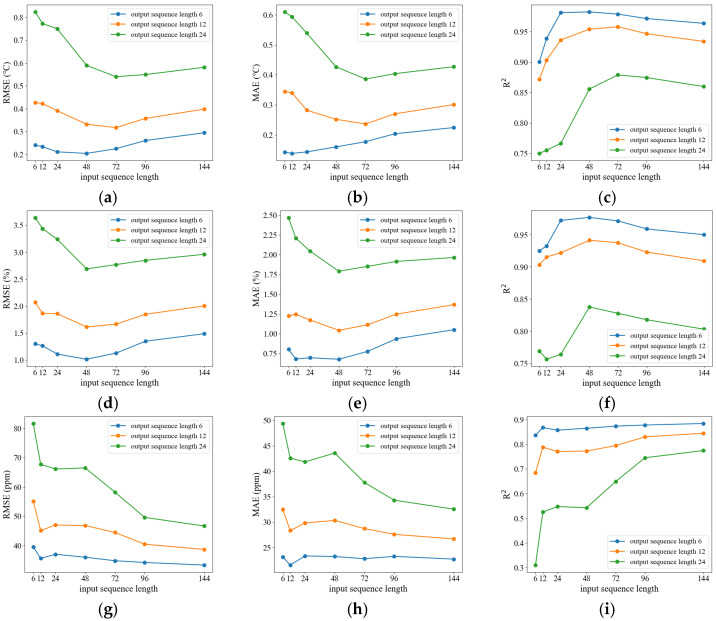
Prediction results under different combinations of input and output sequence lengths. (**a**–**c**) Temperature prediction results, showing the variation of RMSE, MAE, and R^2^, respectively; (**d**–**f**) Humidity prediction results, showing the variation of RMSE, MAE, and R^2^, respectively; (**g**–**i**) CO_2_ concentration prediction results, showing the variation of RMSE, MAE, and R^2^, respectively.

Figure 9 presents the prediction results under different combinations of input and output sequence lengths, where curves of different colors represent different output lengths.

Figure 9 demonstrates that, with a constant input sequence length, increasing the output sequence length results in higher RMSE and MAE values and lower R^2^ values. This trend indicates that model performance declines as the forecasting horizon extends, reflecting increased complexity and uncertainty. When the output sequence length is fixed, increasing the input sequence length generally causes RMSE and MAE to decrease initially, then stabilize or slightly increase. Extending the input sequence length within an optimal range enables the model to capture essential historical information. However, excessively long input sequences may introduce redundant data and noise, which can negatively affect model performance.

Experimental results indicate that, in multi-parameter joint prediction for rabbit houses, prediction targets differ in their reliance on historical data. Selecting a unified input length based solely on average optimal performance may not meet practical production needs. In this context, the relative importance of each indicator was considered. Temperature and humidity are directly involved in environmental regulation, making their prediction accuracy essential for maintaining stable conditions. While CO_2_ concentration remains important, it is not the primary parameter for direct control. Consequently, input length selection prioritized temperature and humidity prediction performance. An input length of 48 is recommended for an output sequence length of 6, and 72 for output sequence lengths of 12 or 24.

### 3.2. Performance Evaluation Experiments

To assess the predictive performance of the proposed model in practical applications, comparative experiments were conducted in accordance with the actual requirements of the rabbit house environmental regulation. Considering that a forecasting horizon of 2 h (i.e., output sequence length of 12) is sufficient for daily control needs, all subsequent experiments were performed with the output length fixed at 12.

RNN, GRU, and LSTM were selected as baseline models for comparison. These recurrent architectures have demonstrated strong nonlinear modeling and temporal memory capabilities, and are widely applied in environmental monitoring and intelligent agricultural control [30,31]. Specifically, RNN, GRU, and LSTM were implemented with a three-layer recurrent structure, 256 hidden units, and the *tanh* activation function.

The experiments were conducted using the Fujian dataset for both training and testing. To ensure fairness, PatchCrossFormer-RHP and the comparative models were configured with identical input and output sequence lengths, as well as the same optimizer (Adam), loss function (mean squared error, MSE), learning rate (0.001), batch size (128), and training epochs (100). The experimental results are reported in Table 6.

Table 6 reports the comparative performance of different models in multivariate forecasting tasks for temperature, humidity, and CO_2_ concentration. Overall, PatchCrossFormer-RHP consistently achieved the best performance across all three environmental parameters. Compared with the “best baseline” (the strongest among the comparative models), the temperature prediction RMSE decreased by 39.1% (0.476 → 0.290), MAE decreased by 40.5% (0.370 → 0.220), and R^2^ improved from 0.905 to 0.963. For humidity prediction, RMSE decreased by 15.4% (1.836 → 1.554), MAE decreased by 17.4% (1.273 → 1.052), and R^2^ improved from 0.923 to 0.956. For CO_2_ concentration prediction, RMSE decreased by 1.7% (39.506 → 38.837), MAE decreased by 14.9% (29.706 → 25.269), and R^2^ reached 0.838, matching GRU. These results verify the effectiveness of the proposed model in multivariate time-series modeling and cross-variable feature extraction.

Both GRU and LSTM consistently outperformed RNN, highlighting the importance of gated structures in modeling both long- and short-term dependencies, as well as in handling non-stationary disturbances. LSTM exhibited better performance than GRU in humidity prediction, while GRU surpassed LSTM in CO_2_ concentration prediction. This indicates that LSTM is more effective at capturing long-term memory in stable, gradually varying processes, whereas GRU, with fewer parameters, offers stronger robustness to noise.

### 3.3. Ablation Study

To quantify the contributions of key modules in PatchCrossFormer-RHP to prediction performance, a series of ablation experiments were conducted. Under the condition that all other structures remained unchanged, each core module of PatchCrossFormer-RHP was removed in turn, and the model was trained and tested on the Fujian dataset. The experimental results are summarized in Table 7.

As shown in Table 7, removing any key module led to a degradation in prediction performance, confirming that patch encoding, the Patch-Global Aggregator, and the cross-attention module all play essential roles in PatchCrossFormer-RHP. When the patch encoding module was removed, the RMSE of temperature, humidity, and CO_2_ concentration increased by approximately 122%, 63%, and 29%, respectively. Meanwhile, the MAE rose by more than 60%, and the R^2^ decreased by over 10%. This suggests that capturing local patterns makes a significant contribution to all variables and forms a critical foundation for enhancing predictive capability.

When the Patch-Global Aggregator was removed, the RMSE and MAE for temperature and humidity approximately doubled, whereas the decline in R^2^ was less than 2%. This suggests that the module makes a minimal contribution to trend capturing but plays a significant role in reducing absolute prediction errors. By aggregating local and global temporal information, it effectively suppresses numerical bias and enhances prediction accuracy.

In comparison, removing the cross-attention module caused relatively smaller performance degradation: the RMSE of temperature increased by 26%, while that of humidity and CO_2_ concentration rose by 15% and 5%, respectively. Although its effect on individual metrics was limited, the cross-attention mechanism plays a crucial role in facilitating feature interaction and information alignment between environmental and auxiliary parameters, thereby enabling the explicit modeling of temporal dependencies among different variables.

In summary, the three key modules each emphasize different aspects of performance and, when combined, jointly ensure the optimal predictive capability of PatchCrossFormer-RHP.

## 4. Discussion

As shown in Section 2.3, significant differences were observed in the environmental parameters of rabbit houses in Gansu, Henan, and Fujian. These differences mainly arose from regional variations in climate conditions, housing structures, stocking densities, management practices, and environmental control systems. To evaluate the generalization ability of PatchCrossFormer-RHP, particularly its adaptability to rabbit houses in different regions, cross-region cross-validation experiments were conducted. The model parameters were first randomly initialized, denoted as PatchCrossFormer-RHP (Init). Under the configurations described in Table 4 and Table 5, PatchCrossFormer-RHP (Init) was then trained separately on the Gansu dataset, the Henan dataset, and a combined dataset of both regions, yielding three pretrained models: PatchCrossFormer-RHP (GS), PatchCrossFormer-RHP (HN), and PatchCrossFormer-RHP (GS + HN). These four models served as the comparative baselines for subsequent analysis.

**Figure 10:**
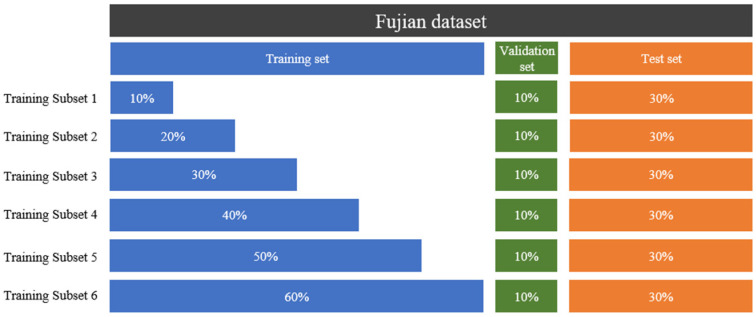
Schematic illustration of dataset partitioning. The percentage values denote the proportion relative to the total number of samples.

The Fujian dataset was divided into training, validation, and test sets with a ratio of 6:1:3. Based on this, the training set was further partitioned into six incremental subsets, as illustrated in Figure 10.

**Figure 11:**
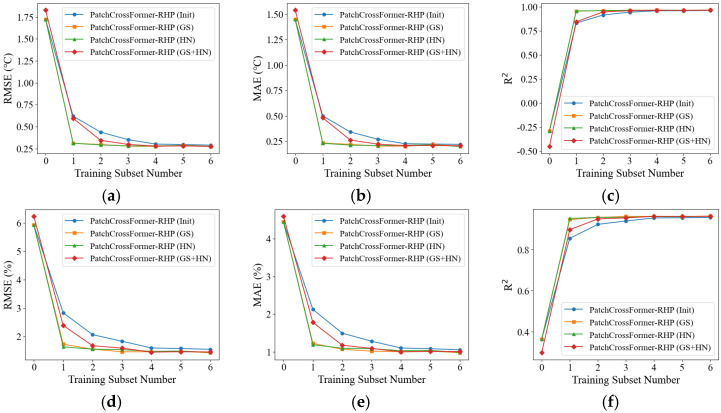

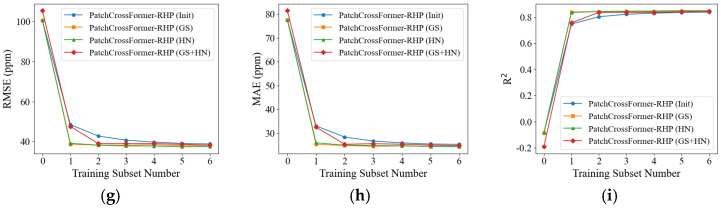
Model performance with varying proportions of the Fujian training dataset. (**a**–**c**) Temperature prediction results, showing the variation of RMSE, MAE, and R^2^, respectively; (**d**–**f**) Humidity prediction results, showing the variation of RMSE, MAE, and R^2^, respectively; (**g**–**i**) CO_2_ concentration prediction results, showing the variation of RMSE, MAE, and R^2^, respectively. The horizontal axis indicates the index of training subsets, where 0 denotes direct testing without training and 1–6 correspond to six incremental subsets containing 10–60% of the Fujian dataset (in steps of 10%).

The four models were first evaluated directly on the Fujian test set to establish baseline performance without training. Subsequently, each model was trained on the six training subsets, with the validation set used for hyperparameter tuning and overfitting prevention during training. After training, the models were evaluated on the unified test set to quantify the impact of training data volume on predictive performance under cross-regional deployment. The experimental results are presented in Figure 11 and Table 7.

When trained on subset 1, all four models demonstrated a significant improvement in predictive performance compared to direct testing on the target dataset without training. Among them, the single-region pretrained models, PatchCrossFormer-RHP (GS) and PatchCrossFormer-RHP (HN), achieved larger and comparable gains, rapidly approaching their respective optimal performance levels. As the training subset size increased, the performance of all four models continued to improve, though at a slower pace. Notably, PatchCrossFormer-RHP (GS + HN) reached near-optimal performance after training with subset 4, requiring less data than the non-pretrained baseline PatchCrossFormer-RHP (Init). These findings suggest that pre-trained models can achieve satisfactory performance in cross-regional deployment with a relatively small amount of training data, and that single-region pre-trained models generally perform better.

The results obtained after training with subset 6 are reported in Table 8.

As shown in Table 8, all three pretrained models outperformed the randomly initialized PatchCrossFormer-RHP (Init) across all evaluation metrics, demonstrating that pretraining improves model performance in the target domain and validating the effectiveness of transfer learning. A comparison of PatchCrossFormer-RHP (GS), PatchCrossFormer-RHP (HN), and PatchCrossFormer-RHP (GS + HN) further reveals that the joint pretraining model (GS + HN) performed worse on all metrics than the single-source pretrained models. This indicates that single-source pretraining is generally superior to joint pretraining in cross-regional tasks. A possible explanation is that the data distributions of Gansu and Henan differ substantially; joint pretraining may cause the model to learn contradictory or overly specific features, thereby weakening its generalization ability.

In summary, pretraining enhances the cross-regional performance of PatchCrossFormer-RHP. In particular, single-source pretrained models achieve the best results, requiring only a small amount of target-domain data during fine-tuning to reach satisfactory performance. This demonstrates the strong adaptability of PatchCrossFormer-RHP across regions, highlighting that an appropriate pre-training strategy can effectively improve both predictive accuracy and transferability in cross-regional scenarios.

## 5. Conclusions

This study proposed a novel PatchCrossFormer-RHP model for predicting temperature, relative humidity, and CO_2_ concentration in rabbit houses, and conducted extensive experiments on Fujian datasets, with pretraining on data from Henan and Gansu to verify cross-regional transferability. Based on systematic analysis, the following conclusions were drawn:

1. PatchCrossFormer-RHP achieved the highest overall accuracy across all environmental parameters, outperforming RNN, GRU, and LSTM. Relative to the strongest baseline, the temperature prediction RMSE and MAE decreased by 39.1% and 40.5%, respectively, while humidity and CO_2_ concentration predictions also exhibited accuracy improvements, with RMSE and MAE reduced by 15.4% and 17.4% for humidity and by 1.7% and 14.9% for CO_2_ concentration. Taken together, these improvements confirm the model’s superior multivariate forecasting capability. The higher accuracy in temperature prediction is of particular practical importance, as temperature is the primary variable governing ventilation control and thermal comfort regulation in rabbit houses.
2. The patch-encoding module contributes most to the model’s ability to capture temporal regularities, as reflected by the largest drop in R^2^ when it was removed. This suggests that patch-based feature decomposition is a key factor in ensuring the model’s high explanatory power and predictive reliability.
3. PatchCrossFormer-RHP demonstrates excellent transferability. When pretrained on datasets from Gansu and Henan, its performance rapidly reached optimal levels after fine-tuning with only 10% of the target region’s data, underscoring its strong potential for practical applications. This performance arises from its patch-based representation and cross-attention design, which together enhance feature alignment and domain adaptability, enabling reliable prediction under varying climatic conditions.

The PatchCrossFormer-RHP model offers both theoretical guidance and practical solutions for intelligent environmental regulation in rabbit houses. In future work, the proposed framework will be integrated into environmental control systems and validated through on-farm experiments. Technical personnel will be able to design control strategies that jointly consider current conditions and predicted trends. The forecasting results can serve as the core component of Model Predictive Control (MPC) for optimization over the prediction horizon, or as auxiliary information for refining threshold-based or fuzzy control rules. This integration enhances the precision and adaptability of environmental regulation, ultimately improving housing stability, animal welfare, and production efficiency. However, the current study is limited by the geographic range of its data sources. Additional validation in more diverse regions and climatic conditions is necessary to comprehensively evaluate generalization and transferability. Future research will expand multi-scenario datasets and pursue lightweight model deployment to improve practicality and application value.

## Figures and Tables

**Table 1:** Characteristics of the rabbit houses.

Farm Location	Geographical Region	Dimensions (m) *	Enclosure Structure
Qingyang, Gansu	Inland, Northwest China	43 × 13 × 3	Color steel panel
Jiyuan, Henan	Inland, Central China	28 × 12 × 4	Brick wall
Nanping, Fujian	Southeastern coastal China	27 × 6 × 6	Brick wall

* Dimensions are expressed as Length × Width × Height (in meters).

**Table 2:** Specifications of sensors used in the rabbit houses.

Sensor Type	Model	Measurement Range	Accuracy	Quantity
Indoor Temperature and Humidity Sensor	RS-WS-N01-2D	−40 °C to 80 °C 0% to 99% RH	±0.2 °C (at 25 °C) ±2% RH (at 60%, 25 °C)	3
Indoor CO_2_ Sensor	RS-CO2-N01	0~5000 ppm	±(50 ppm + 3% F.S.) (at 25 °C)	3
Indoor Ultrasonic Anemometer	RS-CFSFX-N01-2	0~40 m/s	±(0.5 m/s ± 2% F.S.) (at 60% RH, 25 °C)	3
Outdoor Temperature and Humidity Sensor	RS-N01-BYH	−40 °C to 120 °C 0% to 99% RH	±0.5 °C (at 25 °C) ±3% RH (at 60%, 25 °C)	1

**Table 3:** Seven-Day Average Statistics of Outdoor Temperature and Humidity in Three Regions.

Region	Temperature (°C)			Humidity (%)		
	Maximum	Minimum	Diurnal Range	Maximum	Minimum	Diurnal Range
Nanping, Fujian	30.15	23.51	6.64	98.09	74.25	23.83
Qingyang, Gansu	23.15	15.73	7.42	95.03	59.93	35.10
Jiyuan, Henan	29.97	20.45	9.51	97.15	67.26	29.89

**Table 4:** Hardware and software environments for model training and testing.

Computational Environment	Details
Operating system	Windows 10
Programming language	Python 3.7.12
Deep learning framework	PyTorch 1.13.1
CPU	Intel(R) Core(TM) i7-7700K CPU
GPU	Nvidia RTX 1080 GPU
RAM	32 GB

**Table 5:** Training hyperparameters of the models.

Training Hyperparameters	Value
Optimization algorithm	Adam
Batch size	128
Epochs	100
Initial learning rate	0.001
Feature dimension	256
Encoder layers	3

**Table 6:** Comparison of Different Models for Indoor Temperature, Humidity, and CO_2_ Concentration Prediction at 2-Hour Forecast Horizon.

Model	Indoor Temperature			Indoor Humidity			CO_2_ Concentration		
	RMSE (°C)	MAE (°C)	R^2^	RMSE (%)	MAE (%)	R^2^	RMSE (ppm)	MAE (ppm)	R^2^
PatchCrossFormer-RHP	0.290	0.220	0.963	1.554	1.052	0.956	38.837	25.269	0.838
RNN	0.783	0.641	0.744	2.895	2.165	0.810	50.905	39.839	0.731
GRU	0.476	0.374	0.905	1.996	1.374	0.909	39.506	29.706	0.838
LSTM	0.477	0.370	0.904	1.836	1.273	0.923	42.043	32.097	0.816

**Table 7:** Results of ablation experiments on PatchCrossFormer-RHP.

Key Modules			Indoor Temperature			Indoor Humidity			CO_2_ Concentration		
Patch Encoding	Patch-Global Aggregator	Cross Attention	RMSE (°C)	MAE (°C)	R^2^	RMSE (%)	MAE (%)	R^2^	RMSE (ppm)	MAE (ppm)	R^2^
× *	√	√	0.643	0.516	0.827	2.534	1.967	0.854	50.215	40.587	0.738
√ *	×	√	0.619	0.451	0.959	3.473	2.383	0.942	47.616	30.187	0.764
√	√	×	0.365	0.270	0.944	1.780	1.205	0.928	45.771	30.021	0.782
√	√	√	0.290	0.220	0.963	1.554	1.052	0.956	38.837	25.269	0.838

* √ denotes inclusion of the module, and × denotes its removal.

**Table 8:** Prediction performance of models trained with the full training dataset.

Environmental Parameter	Evaluation Metric	PatchCrossFormer -RHP (Init)	PatchCrossFormer -RHP (GS)	PatchCrossFormer -RHP (HN)	PatchCrossFormer -RHP (GS + HN)
Indoor Temperature	RMSE (°C)	0.290	0.276	0.273	0.275
	MAE (°C)	0.220	0.203	0.199	0.207
	R^2^	0.963	0.966	0.967	0.966
Indoor Humidity	RMSE (%)	1.554	1.431	1.447	1.463
	MAE (%)	1.052	0.972	0.988	1.005
	R^2^	0.956	0.962	0.962	0.961
CO_2_ Concentration	RMSE (ppm)	38.837	38.234	37.576	38.230
	MAE (ppm)	25.269	24.343	24.248	24.741
	R^2^	0.838	0.843	0.848	0.843

## Data Availability

The experimental data are original and not convenient for public disclosure. If there is a reasonable request, please contact the authors.
